# Structure Variations and 3D Genome Disruption: Implications in Safety of hPSC-Based Cell Therapy

**DOI:** 10.3390/ijms27104573

**Published:** 2026-05-20

**Authors:** Min Li, Feixue Cui, Tao Na, Qiang Ma, Meichen Guo, Menghe Guo, Kehua Zhang, Shufang Meng

**Affiliations:** 1 Cell Collection and Research Center, National Institutes for Food and Drug Control, Beijing 102629, China; limin@nifdc.org.cn (M.L.); m17320202511@163.com (M.G.); 15135696747@163.com (M.G.); 2 School of Pharmacy, Shenyang Pharmaceutical University, 103 Wenhua Road, Shenhe District, Shenyang 110016, China; cfx16922025@163.com; 3 State Key Laboratory of Drug Regulatory Science, Beijing 102629, China; maqiang16@mails.ucas.ac.cn; 4 Beijing Key Laboratory of Quality Control and Non-Clinical Research and Evaluation for Cellular and Gene Therapy Medicinal Products, Beijing 102629, China; 5 Key Laboratory of the Ministry of Health for Research on Quality and Standardization of Biotech Products, Beijing 102629, China; 6 College of Life Science and Health Engineering, Hubei University of Technology, Wuhan 430068, China

**Keywords:** human pluripotent stem cell, recurrent variation, 3D genome, structure variation

## Abstract

Human pluripotent stem cells (hPSCs) are a promising source for regenerative medicine due to their self-renewal and differentiation capacities. However, genetic instability acquired during reprogramming and in vitro culture presents major safety challenges for clinical translation. Recurrent mutations, especially structural variants (SVs), are of particular concern as they can impair differentiation and increase tumorigenic risk. In this review, we establish and systematically explore a central causal axis: SVs–three dimensional (3D) genome disruption–safety of hPSC-based therapy. We propose that SVs critically compromise therapeutic safety by perturbing the 3D architecture of the genome, leading to pathogenic rewiring of enhancer–promoter interactions. This rewiring, exemplified by “enhancer hijacking” and “enhancer loss,” can aberrantly activate oncogenes or silence tumor suppressors even in the absence of copy number variations. Thus, 3D genome disruption provides a key mechanistic explanation for SV-driven tumorigenic potential and impaired differentiation fidelity in hPSCs. By highlighting this causal axis, our review not only advances the mechanistic understanding of SV-associated risks but also provides actionable insights for the development of more rigorous quality standards for hPSC-based cell therapy products.

## 1. Introduction

**Figure 1:**
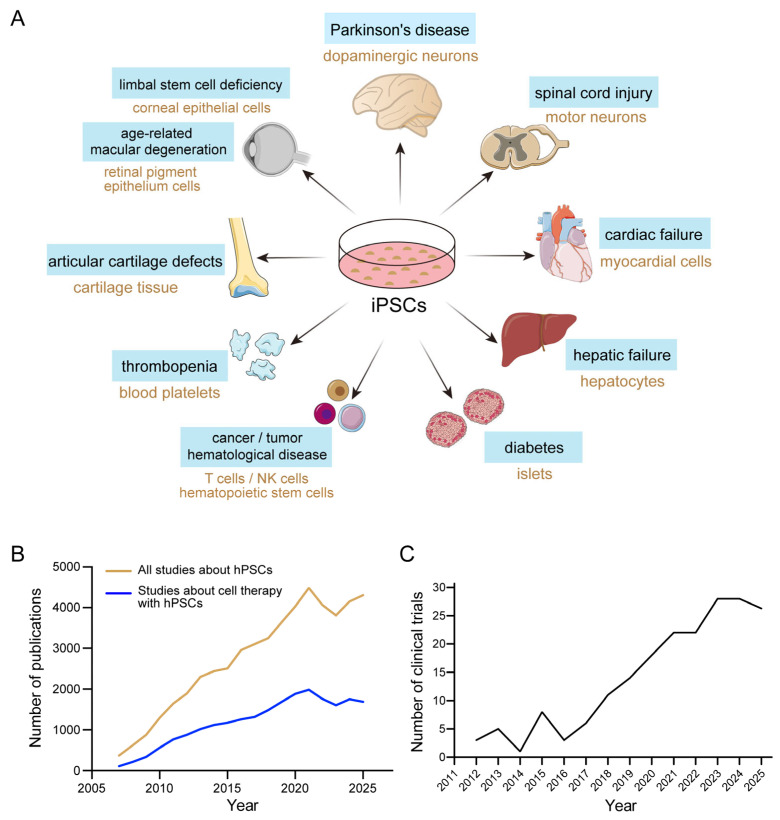
hPSC-derived cell therapy and research. (**A**) Applications of hPSCs in disease treatment. (**B**) Yearly number of articles involved human pluripotent stem cells and cell therapy with hPSCs. (**C**) Yearly number of clinical trials of hPSCs.

Among the various stem cell-based therapeutic strategies, human pluripotent stem cells (hPSCs) hold particular promise as “seed cells” for regenerative medicine because of their extensive proliferative capacity in vitro and potential to differentiate into virtually any cell type. Induced pluripotent stem cells (iPSCs), a type of hPSC discovered in 2007 [1,2], have attracted significant research interest due to the possibility of being reprogrammed from autologous somatic cells, thus eliminating the need for embryonic destruction and associated ethical controversies. In recent years, hPSCs have garnered significant interest for disease treatment, with exploration across various disease areas (Figure 1A). Consequently, both related publications and clinical trials have shown a steady increase (Figure 1B,C). As of December 2024, 115 hPSC-based clinical trials had been approved globally, with over 1200 patients treated. Notably, approximately 60% of these trials utilized iPSCs as the starting cell source [3]. hPSCs are currently being explored for treating a wide spectrum of conditions, including neurodegenerative diseases [4,5], cancer [6,7], ocular diseases [8], and diabetes [9,10], as well as in exosome therapy [11] and assisted reproductive technologies (Figure 1A).

The year 2025 witnessed significant clinical advancements. Several research groups reported clinical outcomes of Parkinson’s disease patients treated with hPSC-derived dopaminergic neurons. These studies demonstrated long-term survival of the transplanted cells in the patients’ brains, significant improvement in motor functions, and a favorable safety profile with minimal adverse effects [12,13,14]. In the field of diabetes, Vertex Pharmaceuticals has published clinical data on their use of hPSC-derived islet cells for the treatment of type 1 diabetes, showing that 83% (10 out of 12) of patients no longer required exogenous insulin therapy by 12 months post-treatment [9]. Furthermore, chemically induced pluripotent stem cell-derived islets have also achieved a functional cure for type 1 diabetes [15]. Despite hPSC-derived cell therapies having shown promising results in small-scale clinical trials or animal models for various diseases, few hPSC-derived cell therapy products have been translated into large-scale clinical applications. A primary concern hindering broader adoption is the potential safety risk associated with the genetic instability of hPSCs. This problem was noticed as early as the first reported clinical trial in 2017 using autologous iPSC-derived retinal pigment epithelium to treat age-related macular degeneration. While one patient achieved a successful therapeutic outcome marked by the sustained survival of the grafted cells for one year, resulting in the arrest of further vision loss, transplantation for another patient was stopped due to the detection of three genomic mutations in the iPSCs of this patient, underscoring the critical importance of safety [16]. In March 2022, a case was reported where a patient developed an immature teratoma following treatment with an iPSC-derived cell product. Notably, this teratoma exhibited higher aggressiveness, greater recurrence potential, and increased cellular heterogeneity compared to typical teratomas, along with more rapid proliferation and expression of the *OCT4* and *SOX2* genes, raising additional safety alarms [17]. These cases highlight the imperative for heightened caution in hPSC-based therapies and the necessity for comprehensive safety assessment of cells prior to treatment. The reprogramming of iPSCs involves profound epigenetic remodeling [18], and the subsequent phases of expansion and differentiation require extensive cell proliferation in vitro [19]. These processes are prone to introducing and accumulating genetic mutations. If these mutations alter cellular phenotypes, they can potentially compromise the differentiation fidelity or the safety of the final cell product. As the application of hPSCs in cell therapy and regenerative medicine expands rapidly, the threat posed by genetic variations accumulated during long-term in vitro culture to the safety of cell therapies becomes increasingly significant. This issue is especially critical now that several hPSC-based products are advancing into Phase III clinical trials and two hPSC-derived therapies for Parkinson’s disease and heart failure have been approved for marketing in Japan, making it essential to understand how mutations in hPSCs impact the safety and efficacy of cellular products.

Structural variants (SVs) are the predominant type of mutation in hPSCs, underscoring the importance of understanding their mechanisms of action. Currently, researchers primarily interpret SVs based on copy number variations in genes within the affected regions. However, this approach overlooks another key mechanism of SVs—the position effect. Therefore, this review proposes the concept of “SV–three-dimensional chromatin structure–safety of cell therapy products,” with a focus on discussing the impact of the position effect of SVs on the safety of cell therapy products. This framework aims to provide a novel perspective for existing safety evaluation systems.

## 2. Recurrent Genomic Alterations in hPSCs

### 2.1. Types of hPSC Genetic Variations

**Figure 2:**
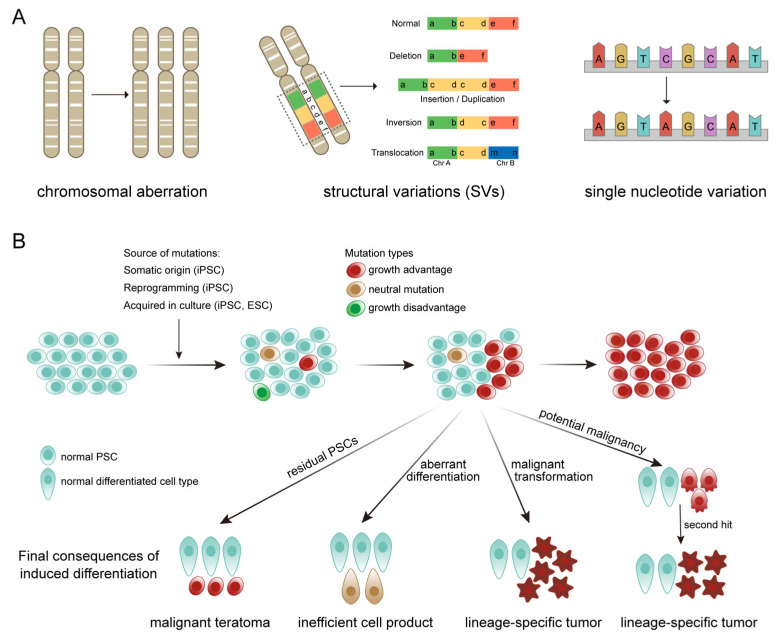
Genetic variants in hPSCs and the consequences. (**A**) Genomic variation types in hPSCs. (**B**) Origins and enrichment genetic variations in hPSCs and their consequences in hPSC-derived therapy.

hPSCs exhibit significant genetic instability during in vitro culture, a phenomenon that becomes particularly pronounced in long-term passaged cells [20]. Additionally, the processes of reprogramming and single-cell cloning during iPSC line establishment may introduce mutations [21,22]. Genetic alterations in hPSCs primarily include whole-chromosome gains or losses, structural variations (SVs) at the sub-chromosomal level, and point mutations (Figure 2A) [23]. SVs, generally defined as variations affecting ≥50 consecutive base pairs, represent a major source of genomic sequence diversity [24]. They include deletions, tandem/insertional duplications (unbalanced SVs, often classified as copy number variations, CNVs), as well as translocations and inversions (balanced SVs with no sequence gain or loss) (Figure 2A) [25,26]. Among the mutations acquired in hPSCs, structural variants are the most prevalent and impactful. Therefore, this review will focus specifically on structural variations in hPSCs.

### 2.2. Mutation Hotspots and Recurrent Genetic Variations

Studies have revealed that mutations in hPSCs are not randomly distributed; instead, certain genomic loci are mutation hotspots, with specific genetic alterations recurring across different hPSC lines [27]. The first report of recurrent mutations in hPSCs dates back to 2004, when Draper et al. identified recurrent amplifications of chromosomes 12 and 17q in human embryonic stem cell lines [28]. Years after the establishment of the first human iPSC line, in 2011, two independent studies provided the first systematic reports of this recurrent mutation phenomenon in iPSCs, demonstrating that amplifications in regions 12p and 20q11.21 could be detected as early as in initial passages [29,30]. Research on both ESCs and iPSCs indicates a high consistency in the spectrum of mutations they acquire. Therefore, for the purposes of this review, mutations in both cell types will be discussed collectively.

A variety of recurrent mutations have been identified in hPSCs. The most common acquired mutations are copy number gains of 1q, 12p, 17q, and 20q, X chromosome segments, and deletions in regions like 10p, 18q, and 22p [31,32]. A report from the International Stem Cell Initiative indicated that approximately 20% of hPSC lines carry an amplification in 20q11.21 region [33]. This specific variant confers a proliferative advantage, allowing mutant cells to outgrow their normal counterparts. Similarly, other recurrent mutations often provide cells with faster proliferation rates or enhanced resistance to apoptosis, leading to the progressive clonal expansion of the mutant population, which can eventually dominate the entire culture system (Figure 2B) [34].

### 2.3. Mutation Occurrence

Mutations in hPSCs are attributable to a combination of intrinsic cellular properties and extrinsic culture conditions.

#### 2.3.1. Intrinsic Cellular Factors

Regarding cell cycle regulation, hPSCs exhibit a shortened G1 phase and a propensity for rapid proliferation, coupled with relatively relaxed cell cycle checkpoints. This unique cell cycle kinetics may allow cells to proceed into mitosis before DNA damage is fully repaired, thereby increasing the risk of chromosomal missegregation or breaks under replicative stress [35,36]. Furthermore, studies have revealed that DNA damage surveillance during the S phase and the decatenation checkpoint in the G2/M phase are more deficient in hPSCs compared to regular somatic cells [37]. This implies that errors during chromatin replication are more likely to arise in hPSCs. Additionally, the highly open chromatin state of hPSCs may render their genome more susceptible to damaging agents [20]. Mutations preexisting in the embryos or donor somatic cells from which PSCs were derived represent an additional source of genetic variations [38,39]. Moreover, during iPSC reprogramming, the overexpression of exogenous transcription factors (such as c-MYC) can induce DNA replication stress [40] and oxidative stress [41], leading to the accumulation of cell lesions, including double-strand breaks. Consequently, the reprogramming process itself can be a source of genetic variation [42].

#### 2.3.2. External Culture Conditions

Environmental culture conditions also significantly impact the genetic stability of hPSCs. Oxygen tension is a critical factor. While standard culture is typically performed at ambient oxygen levels (~20% O_2_), reducing the oxygen concentration to a more physiological range (~5% O_2_) has been shown to mitigate the accumulation of mutations in hPSCs [43]. High oxygen tension elevates intracellular reactive oxygen species (ROS), thereby inducing DNA damage and chromosomal breaks. In contrast, low-oxygen culture reduces oxidative stress, helping to preserve genomic integrity [44]. In the field of cell therapy, hPSCs are typically cultured using feeder-free systems. The extracellular matrices (ECMs) employed during this process can influence the genetic stability of the cells [45]. For instance, vitronectin has been associated with the enrichment of chromosomal abnormalities such as 1q gain [46]. Research by Acevedo-Acevedowe et al. further revealed that the physical properties of ECMs, particularly stiffness, can affect the genomic stability of hPSCs [47]. In a recent study, Changjin Seo et al. developed a novel chemically defined polymer-based substrate capable of maintaining normal karyotypes in hPSC cultures while upregulating the expression of pluripotency-related genes [48]. Collectively, these studies provide critical insights for developing substrates that support the long-term stable culture of PSCs and mitigate genomic abnormalities. Similarly, culture medium composition and methodological practices influence genomic instability. Halliwell et al. indicated that strategies such as using more stable feeder conditions, optimizing enzymatic passaging protocols, and supplementing media with antioxidants or nucleosides can enhance genomic stability to some extent [49]. Their study also proposed that modulating the cell cycle to slow the rapid proliferation rate of hPSCs could be beneficial. For instance, extending the G1 phase using CDK inhibitors provides more time for DNA repair, potentially reducing replication-associated errors [50].

### 2.4. Mutation Accumulation

Following mutation occurrence, selective pressure and clonal expansion are primary drivers of the enrichment of specific variants. If a cell acquires mutation conferring a proliferative advantage, the variant cell will rapidly expand and potentially outcompete the wild-type population [33]. Consequently, mutations that promote cell growth, accelerate the cell cycle, or inhibit apoptosis are positively selected and progressively accumulated. Moreover, they reduces apoptosis triggered by genomic instability [51,52], creating a vicious cycle that promotes further mutation accumulation.

In summary, the dual factors of “mutation generation and selective expansion” drive the emergence and accumulation of genomic instability in hPSC cultures.

### 2.5. Technological Advances in Mutation Detection

The identification of recurrent variations has been profoundly driven by technological advances, achieving a leap from low resolution to single-molecule precision. Early methods with limited throughput, such as traditional karyotyping (G-banding, detecting alterations >5–10 Mb), fluorescence in situ hybridization (FISH), Array Comparative Genomic Hybridization (aCGH) [53] and other high-density DNA arrays [54], have evolved into more accurate and higher-throughput techniques in recent years [55,56]. These include next-generation sequencing (covering whole-genome sequencing, whole-exome sequencing, various epigenomic sequencing [57,58], long-read sequencing [59], single-cell sequencing [60,61]), and platforms like Bionano, which can detect an increasing variety of mutation types [62,63]. The development of new computational tools for SV detection has also made SV identification more efficient and precise [64].

Current insights into the types of mutations in hPSCs, as well as the causes underlying their occurrence and accumulation, have become increasingly sophisticated, alongside improvements in the sensitivity and accuracy of mutation detection. Nonetheless, it remains a major challenge to elucidate the impact of these mutations on cell therapy products and the associated molecular mechanisms. Given that the generation and accumulation of mutations in hPSCs is almost inevitable due to prolonged in vitro culture, the establishment of a more scientifically rigorous safety assessment system for hPSCs can only be achieved by distinguishing “dangerous mutations” that pose true safety risks and by comprehensively deciphering their molecular mechanisms of action. The linear genome-based interpretation framework is far from sufficient to explain the complete phenotypic changes in mutated cells. Therefore, a novel perspective is required for mutation interpretation.

## 3. SVs Compromise Safety of iPSC-Based Cell Therapy Through 3D Genome Disruptions

### 3.1. Structural Variations as Prevalent Risk Factors in hPSCs

SVs represent the most prevalent and impactful class of mutations in hPSCs, and constitute the most common type of recurrent genetic alterations [65,66]. Consequently, understanding the influence of SVs on hPSC phenotypes and their underlying mechanisms is critical for accurately interpreting hPSC genetic mutations and for safely advancing hPSC-derived cell therapies. The threats posed by SVs to the safety of hPSC-based treatments primarily manifest in two aspects: tumorigenic risk [66,67] and impaired differentiation [68] (Figure 2B). Recurrent mutations often confer a growth advantage upon mutant cells. For example, cells harboring a 20q11.21 amplification enhances their resistance to the stresses of culture and provides a growth advantage [69]. In addition to 20q amplification, recurrent copy number gains—including duplications of chromosome 12p and gains of 1q and 17q—are believed to promote cell cycle progression or inhibit apoptosis [25]. While recurrent SVs confer growth advantages to cells, they may simultaneously lay the groundwork for malignant transformation. This is largely attributable to the activation of proto-oncogenes or proliferation-related genes (e.g., *KRAS*, *MYCN*) and the inactivation of tumor suppressor genes (e.g., *TP53*) [70]. Undifferentiated mutant hPSCs within cell therapy products may lead to the formation of malignant teratomas. While differentiated mutant cells may exhibit malignancy and directly form lineage-specific tumors, or transform into malignant cells upon acquiring a second-hit mutation (e.g., *MYCN* amplification), leading to lineage-specific tumorigenesis (Figure 2B). Beyond tumorigenesis, SVs can also compromise the differentiation potential and lineage propensity of hPSCs, often resulting in reduced efficiency in generating specific cell line ages or aberrant cell fate decisions [71] (Figure 2B). For instance, in 2023, Vitillo et al. reported an hPSC line carrying an isochromosome 20 that failed to appropriately generate the three germ layers through spontaneous differentiation, deviating from the normal embryonic developmental trajectory [68]. Similarly, gain of chromosome 1q not only reduces the efficiency of differentiation towards neuroectoderm but also, by upregulating *MDM4* expression, diminishes cellular sensitivity to DNA damage-induced apoptosis [46]. This confers a growth advantage to mutant cells, which persists even after differentiation. Studies on mouse pluripotent stem cells have demonstrated that PSCs carrying specific SVs can form teratomas that are more aggressive and prone to migration to multiple organs, a phenomenon not observed in their genetically normal counterparts [72]. For hPSC-based cell replacement therapies, such alterations in differentiation fidelity, coupled with enhanced proliferative and metastatic potential, can significantly undermine both the efficacy and safety of the final cellular product (Figure 2B).

### 3.2. Parallels Between hPSCs and Cancer Genomic Variations

When SVs elevate the tumorigenic risk of cells, the implications for cell-based therapies become particularly significant. Therefore, this section will focus specifically on the impact of SVs in hPSCs on oncogenic phenotypes and their similarities to SVs observed in tumors. The expert committee of the International Stem Cell Initiative (ISCI) has highlighted in a recent review that many recurrent abnormalities identified in hPSC cultures share similar phenotypic outcomes and molecular mechanisms with genetic alterations found in cancers [32,33]. It raises substantial safety concerns among researchers regarding hPSC-based therapies: even though hPSCs themselves are not cancer cells, the SVs they carry may confer cancer-like behavioral potentials or predispose them to malignant transformation (Figure 2B).

Indeed, many recurrent mutations observed in hPSCs are also prevalent in human tumors, highlighting a concern of genomic alterations in hPSCs (Table 1). This parallelism underscores the potential oncogenic risks associated with specific SVs acquired during hPSC culture. The recurrent amplification of chromosome 12, frequently observed in hPSCs, is a well-documented alteration in tumors such as testicular germ cell tumors and liposarcomas. The isochromosome i(12p) serves as a highly specific marker for testicular germ cell tumors [73,74], while trisomy 12 in lipomatous tumors is associated with malignancy and is utilized as a diagnostic criterion to distinguish well-differentiated liposarcomas from benign lipomas [75,76]. The recurrent 20q duplication in hPSCs is another genetic variant commonly found in human cancers. Approximately 20% of tumors exhibit amplification at the 20q11.21 locus [33]. Notably, 77.8% of gastric adenocarcinoma patients carry 20q amplifications, which are often linked to advanced cancer stages and lymphoma metastasis [77]. Tumors harboring 20q amplifications generally demonstrate greater invasiveness and shorter patient survival. Similarly, 17q amplification—a recurrent mutation in hPSCs—is a frequent genetic abnormality in diverse cancers. A screening of 4429 tumor samples revealed 17q23 copy number gains in 15% of cases, with the highest incidence observed in lung, breast, and soft tissue tumors [78]. Furthermore, partial or complete 17q amplification is detectable in 81% of neuroblastoma patients and serves as a critical indicator for risk stratification, typically placing carriers into intermediate- or high-risk groups [79].

### 3.3. Molecular Mechanisms of SVs

The phenotypic consequences of SVs in hPSCs are primarily mediated through two distinct mechanistic paradigms: dosage effects and non-dosage effects.

#### 3.3.1. Dosage Effects: Direct Impact of Gene Copy Number Alterations

Dosage effects constitute a direct mechanism whereby SVs influence cellular phenotype by altering the copy number of genes or cis-regulatory elements. This can lead to quantitative changes in gene expression levels or result in the direct disruption of coding sequences causing either a loss or gain of gene function.

Previous studies have revealed a striking convergence of dosage-sensitive mutations between hPSCs and cancer cells, frequently involving identical critical genes. For instance, recurrent deletions of key tumor suppressor genes—such as *TP53*, *PTEN*, and *RB1*—and amplifications of proto-oncogenes like *MYC* and *KRAS* are commonly observed in both contexts. Copy number gains on chromosomal arms such as 1q, 17q, and 20q are frequently observed in embryonal tumors and epithelial cancers [56,57,58]. It is hypothesized that the increased gene dosage within these regions promotes tumorigenesis or confers a selective advantage to hPSCs [80,81,82]. Substantial experimental evidence supports the link between gene amplification and phenotypic changes in both hPSCs and tumor cells. For example, amplification of the 20q region, which leads to the overexpression of the anti-apoptotic gene *BCL2L1*, has been demonstrated to enhance cell survival [51,83]. The *TP53* mutation, a well-established driver in numerous cancers, is also subject to natural enrichment as an advantageous mutation in hPSC cultures, highlighting a parallel selective pressure in vitro [84].

#### 3.3.2. Positional Effects Involving 3D Genome Disruption

In contrast to dosage effects, a substantial proportion of SVs exert their phenotypic influence through non-dosage effect mechanisms. It refers to the mechanisms by which SVs affect phenotypes without altering gene copy number, typically positional effects involving 3D chromatin reorganization. This notion is supported by the fact that, in hPSCs, gene expression changes often do not correlate directly with copy number alterations. Transcriptomic data frequently reveal that the magnitude of gene expression changes within amplified regions can be several-fold greater than the gene body copy number variation. Conversely, some genes within amplified regions may exhibit reduced expression [85,86,87]. This paradox suggests the involvement of mechanisms beyond simple gene dosage, pointing to more complex regulatory alterations. The primary proposed mechanism involves the disruption of the 3D genome architecture. SVs can reshape the spatial organization of chromatin, thereby alter its topological structure and lead to the dysregulation of gene expression. A major consequence is the disruption of topologically associating domains (TADs) [88]. This can result in “enhancer hijacking,” where a gene falls under the control of an inappropriate enhancer, or “enhancer loss,” where a gene is disconnected from its native regulatory element. Such positional effects can profoundly alter gene expression patterns without necessarily changing the gene’s copy number. Given their potential to rewire broad regulatory networks, non-dosage effects may exert more extensive and dramatic impacts on cellular phenotype than dosage effects alone.

**Figure 3:**
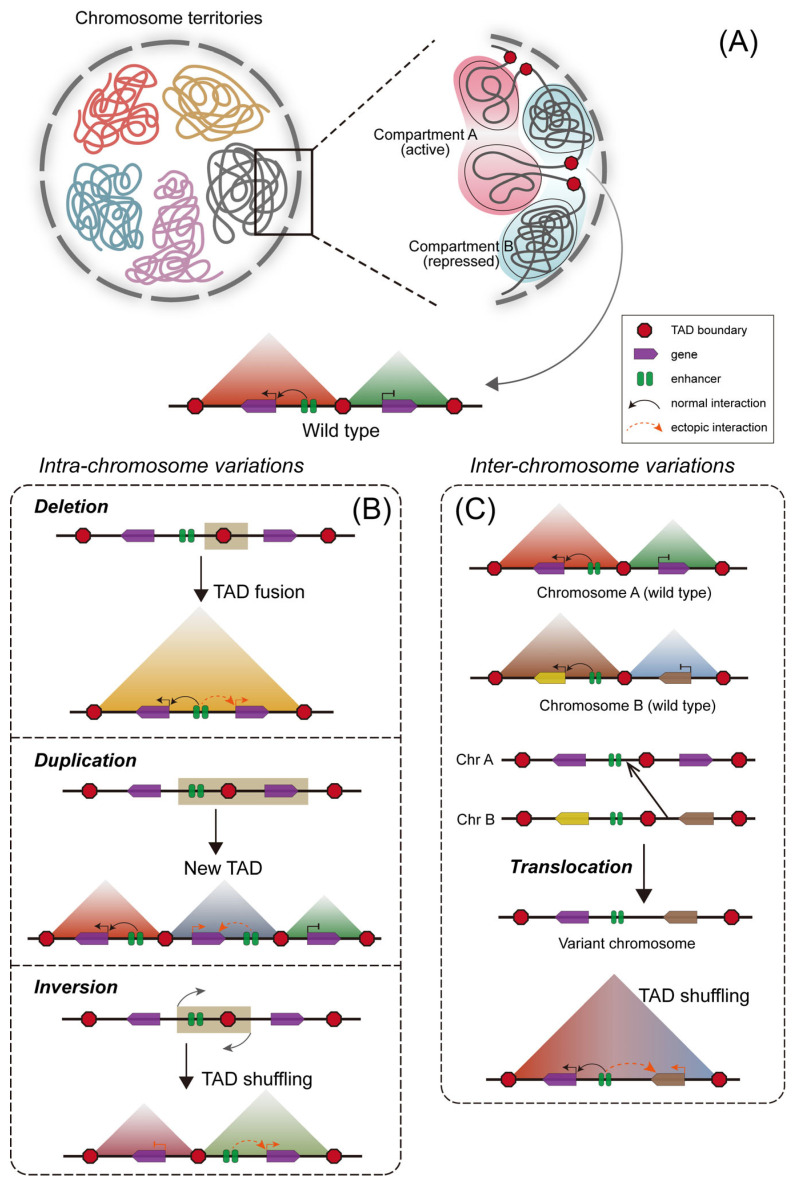
Genome organization and dysregulation of gene expression by rearrangements of TAD. (**A**) 3D organization of genome. From large to small scales: chromosome territories, A/B compartments, TADs. (**B**,**C**) TAD rearrangements and gene misexpression derived by SVs. Wild-type interactions are shown as solid black lines, while ectopic interactions are represented by red dashed lines. Normally gene expressions are depicted by black arrows, while aberrant gene expression is represented by red arrows.

The genome within the nucleus exhibits a hierarchical organization, underpinned by an ordered architecture at multiple scales. At the chromatin level, each chromosome occupies a distinct territory within the nucleus with minimal intermingling, a concept known as chromosome territories (Figure 3A). At a smaller scale, the nucleus is partitioned into two principal compartments with distinct physical properties and functional states: the transcriptionally active A-compartment and the more repressed B-compartment. Each compartment comprises several megabase-sized regions of varying sizes (Figure 3A) [89,90]. A critical level of organization is the TAD (Figure 3A). TADs are sub-megabase to megabase-sized regions wherein DNA sequences interact with each other far more frequently than with sequences outside the domain [91,92]. Genomic loci within the same TAD are spatially much closer to each other than loci in different TADs, even if the linear genomic distance is similar, thereby promoting functional interactions [91]. At the finest scale, the genome is organized into numerous chromatin loops, spanning hundreds of kilobases. These loops are typically formed by the physical approximation of cis-regulatory elements (such as enhancers) and gene promoters, mediated by transcription factors and architectural proteins like CTCF and cohesion [93,94]. This multi-layered, hierarchical organization—from chromosome territories to compartments, TADs, and loops—is fundamental for the precise regulation of gene expression and cellular function. Hi-C is currently the most commonly used method for capturing 3D chromatin architecture, providing data that allow the construction of global chromatin interaction maps. Other more targeted techniques—such as Capture-C [95], HiChIP [96], and ViCAR [97]—as well as low-input Hi-C and single-cell Hi-C [98], which require minimal cell numbers, have also been developed. Together, these technologies enable us to understand how SVs alter chromatin 3D architecture and gene regulatory networks. Alterations in the 3D chromatin architecture, particularly at the level of TADs, can lead to pathogenic gene misregulation, as discussed in the following sections.

### 3.4. 3D Genome Disruption and Pathogenic Gene Misregulation

Within the three-dimensional chromatin architecture, TADs play a critical role in gene regulation by compartmentalizing the genome [99]. They facilitate appropriate interactions between genes and their cis-regulatory elements while insulating against aberrant contacts with inappropriate regulators. The disruption of TAD boundaries by SVs is a significant mechanism leading to pathogenic gene misregulation. When an SV deletes or rearranges a TAD boundary, it can cause genes to interact with ectopic enhancers (enhancer hijacking) or lose contact with their native enhancers (enhancer loss), thereby leading to abnormal gene expression levels [100]. Specifically, certain deletions can fuse two adjacent TADs, promoting illegitimate contacts between otherwise insulated genomic elements and thereby leading to enhancer hijacking (Figure 3B). Duplications of a genomic fragment containing a TAD boundary can result in the formation of a neo-TAD, which integrates regulatory elements and genes from distinct original TADs and ultimately results in enhancer hijacking (Figure 3B). Similarly, inversions or interchromosomal translocations that involve TAD boundaries can profoundly reorganize TADs architecture, disrupting existing interactions and generating aberrant ones, thereby leading to enhancer hijacking and enhancer loss (Figure 3B). Moreover, large segmental gains and translocations have the potential to reshape chromosome territories and A/B compartment status, thereby driving widespread reorganization of three-dimensional chromatin interactions and culminating in complex outcomes, including enhancer hijacking and enhancer loss. These SV-induced aberrant contacts significantly alter gene expression patterns without changing the gene’s copy number [101]. For instance, studies in glioblastoma have shown that SVs spanning different TADs disrupt the native TAD structure, causing gene expression abnormalities; some SVs can even subject a target gene to both enhancer hijacking and enhancer loss simultaneously [102]. Multiple other studies confirm that cancer cells frequently utilize structural rearrangements to achieve enhancer hijacking or loss, thereby activating oncogenes or silencing tumor suppressor genes. A classic example is from T-cell acute lymphoblastic leukemia (T-ALL), where an SV deleted a CTCF-mediated TAD boundary, leading to the promoter of oncogene *MYC* interacting with a super-enhancer and inducing *MYC* aberrant overexpression [103]. Another study of 18 T-ALL patients identified 34 new translocation events and 44 translocation-mediated neo-loops. The 3D genome alterations lead ectopic expressions of multiple oncogenes through enhancer hijacking in 78% (14/18) of cases [104]. Research on 50 cancer cell lines further demonstrated that multiple SVs in tumors drive oncogenesis through enhancer hijacking mediated by alterations in 3D chromatin structure [72].

Beyond cancer, studies on genetic diseases also show that SVs causing ectopic gene expression can lead to differentiation defects and developmental disorders. A typical example involves structural changes at the boundary of the TAD containing *EPHA4*, which drive abnormal interactions between enhancers and genes like *WNT6*, *IHH* or *PAX3*, initiating expression that results in abnormal limb skeletal development [105]. In a case of heterotopic ossification, an 820 kb fragment from chromosome 2 was inserted into Xq26.1. In mutant cells, this led to the formation of two new TADs and abnormal interchromosomal interactions between chromosomes 2 and X. The *ARHGAP36* gene, located within a novel TAD, showed dramatically upregulated expression, activating downstream bone development pathways and causing cells to differentiate into bone cells, potentially explaining the disease etiology [106].

**Table 1:** Pathogenic SVs in hPSCs: from 3D genome disruption to safety risk.

SV	SV in Patient-Derived iPSCs	Culture-Acquired SVs in hPSCs	SV Identified in Diseases	3D Genome Disruption Evidence	Risk
trisomy 12		✓ [107,108]	✓ (tumor) [109]	✓ [110]	high tumorigenic risk
20q duplication		✓ [33,111]	✓ (tumor) [112,113]		high tumorigenic risk
17q amplification	✓ [114]	✓ [86,115]	✓ (tumor) [116]	✓ [117,118,119]	high tumorigenic risk
1q amplification		✓ [120,121]	✓ (tumor) [122,123]	✓ [124]	high tumorigenic risk
2p amplification		✓ [107]	✓ (tumor) [125,126]	✓ [127]	high tumorigenic risk
t(6;18)(q22.31;p11.22)	✓ [128]		✓ [128]	✓ [128]	lineage-specific failure
der(X)dir ins(X;9)(q27.1;p24.3) and der(X)inv ins(X;3)(q27.1;p14.2)	✓ [129]		✓ [129]	✓ [129]	lineage-specific failure
t(3;8)(q26.2;q24)	✓ [130]		✓ (tumor) [130]	✓ [130]	high tumorigenic risk
trisomy 21	✓ [131]		✓ [131]	✓ [131]	lineage-specific failure
inv(6)(p24.3;q16.2)	✓ [132]		✓ [132]	✓ [132]	lineage-specific failure

By analogy, if an SV acquired by an hPSC disrupts the normal 3D genome architecture, it could induce similar regulatory abnormalities, potentially leading to malignant transformation or differentiation defects. Research indicates that certain recurrent variants in hPSCs functionally resemble those found in tumors: in both contexts, they enhance cell survival and self-renewal, thereby supporting clonal expansion or, in cancer, uncontrolled proliferation. Therefore, mechanistically, structural variations in hPSCs could also increase tumorigenic risk through 3D genome disruption. While the important role of chromatin 3D structure in hPSC differentiation and in somatic cell reprogramming to iPSCs has been extensively studied, direct evidence linking SVs to hPSC phenotype, particularly tumorigenicity, via chromatin 3D structural changes remains relatively scarce. Future research focusing on this mechanistic link is crucial for a comprehensive risk assessment of hPSC-based therapies.

## 4. 3D Genome Organization in Human Pluripotent Stem Cells

The functional state of hPSCs is closely related to the 3D architecture of the genome within the nucleus [133,134]. In recent years, with advancements in chromatin conformation capture technologies, researchers have unveiled the unique characteristics of chromatin spatial organization in hPSCs and its role in gene regulation [135,136]. The 3D chromatin architecture of hPSCs plays a pivotal role in the successful reprogramming to iPSCs, the maintenance of pluripotency, and the regulation of their differentiation potential [137,138]. Therefore, when SVs disrupt the normal 3D chromatin architecture, they are likely to induce phenotypic changes in hPSCs.

### 4.1. 3D Genome in hPSCs

The distinct 3D genome of hPSCs is closely related to their gene expression profiles and pluripotent state [139,140]. The spatial conformation of chromatin directly regulates genomic interactions by physically bringing regulatory elements into proximity, a mechanism essential for orchestrating enhancer–promoter communication and precise gene regulation. In hPSCs, key genes that maintain pluripotency, such as *OCT4/POU5F1*, *SOX2*, and *NANOG*, form a highly interconnected regulatory network. These gene loci often engage in physical interactions through chromatin loops, bringing distantly located enhancers and promoters into close proximity, thereby enabling coordinated regulation of gene expression [139,141]. de Wit et al., using 4C technology, discovered that in hESCs, the *NANOG* gene locus forms an “interactome” with other pluripotency regulatory elements, including the *NANOG* promoter, its distal enhancer, and binding sites for other key factors such as *OCT4*, *SOX2* and *c-MYC* [142]. These elements, mediated by the mediator complex and structural proteins like CTCF and cohesin, assemble into a physically proximal complex [143]. This 3D configuration allows pluripotency genes to be synchronously activated or silenced, ensuring the maintenance of the stem cell state. Upon the initiation of differentiation, similar interactomes undergo reorganization; the previously clustered pluripotency genes separate from each other, giving rise to new lineage-specific interactions [144]. In 2024, Li et al. reported differences in the 3D chromatin architecture of naïve-state versus primed-state hPSCs, using single-cell imaging and sequencing. The naïve-state nucleus displayed a more compartmentalized architecture, while the primed-state genome was characterized by a more interwoven and mixed organization [145]. Thus, the 3D genome structure of hPSCs facilitates the coordinated control of pluripotency gene expression through fine-tuned regulation of the enhancer–promoter interaction network and plays a crucial role in pluripotent cell state transitions. By disrupting these critical three-dimensional structures, SVs may alter the pluripotency status of hPSCs, resulting in compromised pluripotency and thereby limiting their utility in cell therapy applications.

### 4.2. 3D Genome Dynamics During Cellular Reprogramming and Differentiation

**Figure 4:**
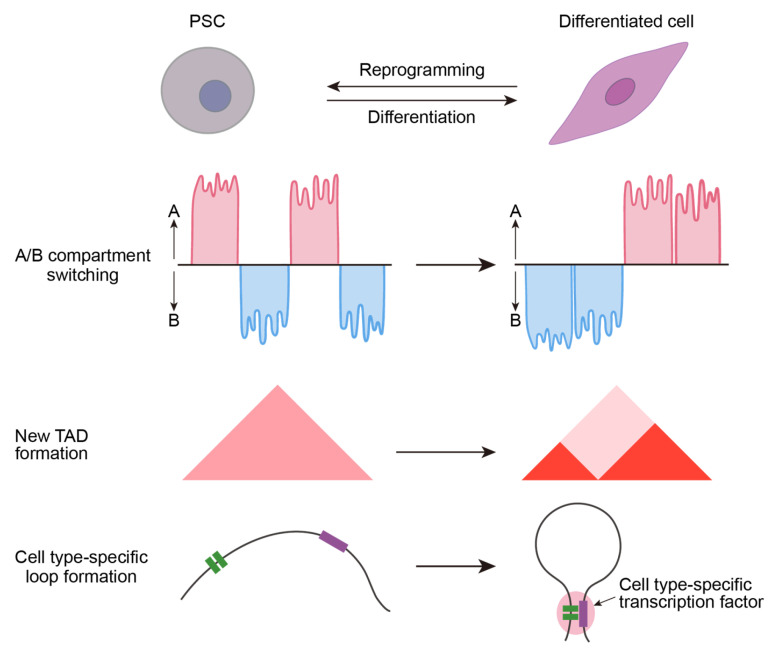
3D genome rewiring during reprogramming and differentiation.

The plasticity of the 3D genome allows cells to rapidly reconstruct the necessary regulatory framework during fate transitions. During the reprogramming of somatic cells to iPSCs, the higher-order structure of chromatin undergoes drastic reorganization, which is essential for activating the pluripotency gene network and silencing somatic gene expression [146]. Studies indicated that in the early stages of reprogramming, somatic-specific chromatin interactions, such as enhancer–promoter linkages, are gradually disrupted, and pluripotency gene loci begin to establish contacts with new regulatory elements [147] (Figure 4). Hi-C analysis of hPSC by Dixon et al. revealed that reprogramming involves global switching of chromatin A/B compartments: many pluripotency genes shift from the repressive compartment B to the active compartment A, while originally differentiation-associated genes are relocated to a more condensed chromatin environment [148]. Factors like OCT4 used in reprogramming promote the formation of chromatin loops, pulling distal enhancers towards pluripotency gene promoters, thereby activating these previously silent genes [149]. Simultaneously, reprogramming triggers local reorganization of TADs and the formation of new chromatin loops (Figure 4), enabling core stem cell factors such as *OCT4*, *NANOG*, and *SOX2* to initiate efficient transcription. Research by Apostolou et al. [143] further indicated that the 3D conformation of some loci may not be completely reset after reprogramming—local chromatin folding may retain aspects of the original somatic state, a phenomenon termed “incompletely reprogrammed sequences.” These regions often correspond to somatically expressed genes that show residual expression, suggesting that 3D structural “memory” may lead to transcriptional memory even when iPSCs have globally reached a pluripotent state [146]. This phenomenon explains why gene expression profiles differ among iPSC clones—some clones may be more thoroughly reprogrammed at the chromatin structure level, thus turning off more source cell genes. In summary, the dynamic changes in the 3D genome during reprogramming play a key role in shaping the epigenetic and transcriptional network of hPSCs: only when chromatin topology is appropriately rearranged can pluripotency genes be stably activated and somatic lineage genes reliably silenced.

When hPSCs are directed to differentiate into specific lineages, their chromatin interaction atlas is reconstructed to adapt to new transcriptional programs. During differentiation, some chromatin contacts that were active in the pluripotent state weaken or disappear, while new cell type-specific chromatin interactions are gradually established (Figure 4). This reorganization facilitates the stable pairing of enhancers with key developmental gene promoters and can be accompanied by the formation of new TAD boundaries to insulate distinct gene regulatory modules.

Experimental evidence shows that in various systems, such as neural and cardiac differentiation, the chromatin interactome undergoes multi-level reorganization (Figure 4). For example, during skeletal muscle differentiation, loci related to muscle development form new enhancer–promoter loops, while pluripotency-related interactions disintegrate [150]. During in vitro neural differentiation and in vivo embryonic development, chromatin also exhibits multi-scale 3D remodeling [147]. These structural changes have clear biological significance: they ensure lineage-specific genes are expressed on demand while the pluripotency network is shut down. The binding patterns of 3D structural maintenance proteins like CTCF and cohesin change during differentiation, leading to the dissolution of some pre-existing chromatin loops and the emergence of new ones [151]. Overall, cell fate decisions require corresponding reorganization of chromatin topology as support [152]. Relevant studies prove that epigenetic changes, including 3D chromatin structure, during differentiation precede changes in gene transcription, further demonstrating the important role of 3D chromatin structure in cell differentiation [153]. Proper 3D genome reorganization is a prerequisite for accurate cell fate specification. Its impairment can result in either incomplete or aberrant differentiation. Therefore, SVs that impair the proper formation of differentiation-associated three-dimensional chromatin structures, or hinder the timely dissolution of pre-existing architectures, can lead to differentiation failure of cell therapy products and may pose a heightened safety risk, including increased tumorigenic potential.

### 4.3. Functional Implications of 3D Genome Disruption by Structural Variations

3D chromatin architecture functions both as a scaffold for the pluripotent state and as an active participant in cell fate determination in hPSCs. Given its pivotal role, the three-dimensional genome must be taken into account when interpreting the impact of SVs. Although there is currently no direct research on whether recurrent SVs in hPSCs affect their phenotype through chromatin 3D structure, studies using patient-derived iPSCs or gene-edited cells modeling patient mutations have revealed the important role of chromatin conformation changes caused by SVs in hPSCs. Deletion of *MEF2C* is common in neurodevelopmental disorders (NDDs), leading to differential expression of genes related to neurodevelopmental pathways and synaptic function, as well as reduced neuronal synaptic activity. Researchers used CRISPR technology to delete the centromere-proximal region of the TAD containing *MEF2C*, finding that it led to downregulation of *MEF2C* expression and weakened electrophysiological activity in knockout cells, effects similar to directly knocking out the gene [154]. In iPSCs reprogrammed from peripheral blood mononuclear cells of a patient with central iris hypoplasia carrying a balanced translocation t(6;18)(q22.31;p11.22), the original TAD was disrupted, and a new TAD formed on the derivative chromosome, causing an enhancer cluster to contact the APCDD1 promoter, creating abnormal interactions and upregulating *APCDD1* expression. Overexpression of this gene may interfere with the Wnt signaling pathway affecting iris development, potentially explaining the familial disease cause [128]. In patients with hereditary retinal dystrophy, researchers discovered two interchromosomal translocations, der(X)dir ins(X;9)(q27.1;p24.3) and der(X)inv ins(X;3)(q27.1;p14.2), associated with 3D genome disruption. They reprogrammed fibroblasts from mutation carriers and normal individuals into iPSCs and differentiated them into retinal organoids and retinal pigment epithelial cells. They found that both mutations, through TAD reorganization, caused the originally silent *LINC00632* to make ectopic contact with an enhancer, initiating expression, subsequently leading to tissue-specific dysregulation of *LINC00632* and *CDR1as/ciRS-7*. This tissue-specific gene dysregulation is considered the pathogenic mechanism of retinal degeneration in the two patients [129]. Beyond typical Mendelian diseases, 3D structural alterations related to cancer in hPSCs have also been reported. In 2024, Nakamura reported their study on iPSCs from a myelodysplastic syndrome (MDS) patient with a t(3;8)(q26.2;q24) translocation. They differentiated the patient’s iPSCs into hematopoietic progenitor cells to simulate the MDS pathogenesis process, successfully recapitulating the patient’s *MECOM* expression change pattern. This was due to chromatin 3D structure changes caused by the translocation connecting the *MECOM* promoter and a *MYC* super-enhancer, leading to overactivation of *MECOM* expression [130]. Notably, this type of mutation might be overlooked in routine transcriptome analyses as it only involves spatial chromatin conformation changes without affecting the gene coding sequence.

Although current evidence for SVs affecting hPSC phenotype and cell product safety through 3D chromatin structure is still limited, existing research results indicate the important role of 3D chromatin structure as a bridge between mutation and phenotype. Given the critical role of a correct three-dimensional chromatin architecture in processes such as pluripotency maintenance and cell differentiation of hPSCs, any SV that disrupts the normal 3D chromatin structure of hPSCs may lead to phenotypic abnormalities, differentiation failure, or even malignant transformation, thereby compromising the safety and efficacy of hPSC-derived cell therapy products. The development of an assessment model that considers linear and 3D genomic effects in a tissue-specific perspective will facilitate improved identification of high-risk mutations (Table 1).

## 5. Summary and Perspective

The safety concern surrounding hPSC-based therapies is fundamentally an issue of genomic stability. hPSCs undergo extensive in vitro expansion, and iPSCs face additional procedures including reprogramming and clonal selection, making mutation acquisition inevitable. Consequently, the accurate identification of risk-increasing variations constitutes a critical imperative for risk assessment. Current mutation detection technologies are progressively improving, enabling rapid and precise identification of mutations within cell culture systems, including low-frequency variants [155]. However, the molecular mechanisms by which these mutations influence cell phenotype and potentially introduce safety risks into therapeutic products remain poorly understood [156]. With growing knowledge of the non-coding genome, researchers increasingly recognize that genetic variations impact cellular biology not only through gene dosage effects but also, significantly, via disruption of the 3D genome [157].

SVs are the most common recurrent mutations in hPSCs. Should these SVs confer oncogenic potential to mutant cells, this would pose a critical threat to the safety of derived cell therapy products. Notably, researchers have observed a substantial overlap between SVs found in hPSCs and those in tumors [19,34], strongly indicating the need for heightened scrutiny of SVs in hPSCs, particularly those resembling oncogenic variants. Admittedly, important differences exist between hPSCs and tumor cells. SV acquisition in hPSCs occurs within a controlled artificial environment, typically enriching only a small subset of driver mutations. In contrast, tumor cells undergo selection within a complex in vivo microenvironment, accumulating multiple layers of alterations and acquiring malignant phenotypes such as uncontrolled proliferation and invasion [158]. Furthermore, mutations in hPSCs are often related to growth advantage, whereas tumor cells additionally acquire capabilities like immune evasion and angiogenesis promotion. Nevertheless, the acquisition of relevant mutations by hPSCs significantly increases the tumorigenic risk of the cell product. Consequently, comparing the mutational landscapes of hPSCs and tumor cells, and understanding the molecular mechanisms of these mutations, will facilitate the establishment of superior quality control strategies for hPSCs.

SVs have been demonstrated in various cancers to drive tumorigenesis by disrupting 3D chromatin structure, leading to ectopic overexpression of oncogenes or silencing of tumor suppressor genes [159]. And 3D chromatin structure plays a crucial role in hPSCs. It is not only critical for maintaining pluripotency but also instrumental in driving both reprogramming and differentiation [18]. Therefore, it is highly probable that SVs in hPSCs also influence cell phenotype, including tumorigenicity and differentiation potential, through alterations in the 3D chromatin structure. Multiple studies have provided evidence supporting the link between 3D structural changes and functional phenotypes. In summary, advances in understanding the 3D chromatin architecture of hPSCs have not only deepened our insights into pluripotency and differentiation regulation but also provided new perspectives for stem cell applications and regenerative medicine.

With technological advances, the cost of chromatin conformation capture assays has decreased significantly, making it feasible to apply Hi-C sequencing to the quality control of therapeutic hPSCs. Hi-C data enable the construction of global chromatin interaction atlas, which can assist researchers in identifying SVs. Moreover, as the 3D chromatin architecture serves as a critical bridge between the genome and cellular phenotypes, Hi-C data can be used not only for safety assessment but also for evaluating the pluripotency status and differentiation propensity of hPSCs, thereby facilitating a comprehensive characterization of hPSC states. At critical checkpoints such as the establishment of hPSC master cell banks, working cell banks for cell therapy, and prior to transplantation of cell products, Hi-C sequencing can be employed in quality control and release testing. For precious cell products such as NK cells, low-input Hi-C is a preferred option to minimize cell input requirements. By designing specific probes for recurrent SVs and oncogenes, Capture-C can generate higher-resolution, targeted maps of regional chromatin 3D interactions. This strategy is particularly useful for assessing whether oncogenes have hijacked active enhancers, thus enabling the evaluation of tumorigenic risk. The interaction profiling of common risk regions, including oncogenes, could be further developed into a commercialized detection method and incorporated into routine monitoring. Once sufficient data have been accumulated in hPSC, deep learning tools can be integrated to establish a predictive model linking SVs, chromatin 3D architecture, and safety phenotypes. By inputting SV information, the model could predict the impact of SVs on chromatin 3D architecture and their potential functional consequences under different contexts (e.g., differentiation into distinct cell lineages).

To mitigate the negative impacts of genomic instability in human pluripotent stem cells (hPSCs), multifaceted control strategies are being actively implemented and developed. One approach focuses on prevention—optimizing culture conditions to reduce variant generation and selection, including the aforementioned low-oxygen culture, nucleoside supplementation, and strict control of passaging density [20,43]. The other approach involves early detection and intervention, utilizing sensitive molecular detection methods to identify abnormal clones promptly for removal. With advancements in sequencing technologies, whole-genome sequencing and single-cell sequencing are increasingly being applied to hPSC quality control, enabling the detection SVs at the subclonal level for higher precision monitoring. Notably, establishing “zero mutations” hPSCs is an unrealistic goal, and approximately half of SVs in human genome are non-pathogenic [160]. Thus, establishing a unified standard for the evaluation of genetic stability is an urgent issue that needs to be addressed in the field. This is particularly pressing for ex vivo gene-edited hPSC products, where off-target effects or unintended structural rearrangements introduced during genome editing occur frequently. Currently, international organizations are actively promoting the establishment of reference standards and comprehensive evaluation frameworks. In 2026, the International Society for Stem Cell Research (ISSCR) released key quality control testing methodologies for the genetic characterization of hPSCs, including karyotyping, digital PCR, WES, and WGS, among others [161]. However, it remains challenging to effectively assess the safety risks of mutations detected by these methods in hPSCs, particularly those located in non-coding regions. Furthermore, no unified standards exist to determine which mutated cells may be permissible for subsequent cell therapy and which should be prohibited from such applications. 3D genome analysis can serve as a complementary method that bridges current genetic stability assays (such as karyotyping and WGS) and functional assessments (such as teratoma formation and differentiation assays). While genetic assays identify the presence of SVs and functional assays assess their downstream consequences, 3D genome analysis provides mechanistic insight into how SVs exert their effects—for example, by disrupting TAD boundaries or enabling enhancer hijacking. Thus, integrating 3D genome analysis into the QC workflow offers a missing link between genotype and phenotype and can provide a novel dimension and unprecedented important information for hPSC quality assessment. This integration would support the distinction between high-risk and benign mutations and the establishment of a more comprehensive safety evaluation system.

In summary, research on hPSC genetic instability has deepened our understanding of its patterns and mechanisms. Incorporating chromatin 3D structure into safety assessments will help advance more robust monitoring and control strategies. These practices are beneficial to enhance the safety of hPSC-derived cell therapies, ensuring their reliable application in both research and clinical settings.

## Data Availability

No new data were created or analyzed in this study. Data sharing is not applicable to this article.
